# Who Signs Up for Singlehood and Romantic Relationship Studies? Examining Volunteer Bias in Online Recruitment

**DOI:** 10.1177/01461672251390020

**Published:** 2025-12-16

**Authors:** Elaine Hoan, Yoobin Park, Geoff MacDonald

**Affiliations:** 1 University of Toronto, Ontario, Canada; 2 University of California, San Francisco, USA

**Keywords:** volunteer bias, framing effects, singlehood, romantic relationships

## Abstract

Research involving self-report methods risks volunteer bias, which can undermine validity by attracting particular participant types. What is the risk of such bias when participants choose studies on online recruitment platforms? The current study (Study 1: *N* = 1,595; *M*_age_ = 28.45; Study 2: *N* = 2,777; *M*_age_ = 31.18) examined volunteer bias in online studies, using the context of recruiting individuals for singlehood and romantic relationship research. Participants were recruited via *Prolific* for a study about “people’s lifestyles,” or “singlehood [or romantic relationships] and people’s lifestyles.” We assessed and compared their demographics, individual differences, feelings about singlehood/partnership, and well-being. No consistent differences emerged across recruitment framings, suggesting that advertisement wording did not selectively attract distinct *Prolific* participants. These data support one aspect of the validity of online singlehood and relationship research, suggesting that low-effort studies conducted on online platforms may suffer less from volunteer bias than other research recruitment strategies.

Beginning in 2010, a growing concern emerged among researchers surrounding the reliability and validity of scientific results, particularly in the field of psychology. Concerns centered on the possibility that various widely implemented practices could result in consequences like highly inflated false-positive error rates in the psychological literature (Shrout & Rodgers, 2018). An important response to the growing recognition of these problems was the building of the Open Science movement which advocates for making the scientific research process transparent and accessible (Open Science Collaboration, 2015). While improving the replicability of studies has been one important focus, increasing attention is also being given to making improvements when it comes to validity (Flake et al., 2017).

Among various validity concerns is whether unrepresentative convenience samples limit the real-world applicability of research such as findings based on college samples not replicating in broader populations (Peterson & Merunka, 2014). One source of unrepresentative samples is *volunteer bias*—when particular individuals who differ from population averages are more likely to opt into a study (Martin & Marcuse, 1958; Rosenthal & Rosnow, 2009; Waters & Kirk, 1969). Past work has shown that topic salience, or how important a survey topic is to an individual, predicts higher likelihood of participation (Vaughan-Johnston et al., 2024; Zillmann et al., 2014). Volunteer bias could lead researchers to mistakenly generalize findings to a broader population when the results actually apply narrowly to the subgroup attracted to that study. Existing work has begun to clarify conditions that would generate volunteer bias. Such bias appears especially pronounced for sensitive topics, such as sexuality, where study framing has been shown to select for participants with higher rates of sexual abuse histories (Anderson et al., 2022; Regan, 2008). Likewise, recruitment into more intensive, high commitment studies, like in-person interviews or longitudinal studies (Barton et al., 2020; Park et al., 2021), might generate volunteer bias. Less clear, however, is whether volunteer bias emerges in less intensive methods, such as the commonly employed online self-report survey method.

Online studies may be vulnerable to volunteer bias, particularly through framing effects. Online platforms are attractive to researchers as an efficient way to collect data. Over the past few decades, online recruitment has surged in psychology and social sciences with the rise of platforms like *Mturk* and *Prolific* (Buhrmester et al., 2018). However, online surveys allow participants to browse through multiple postings and select studies based on the title and description, potentially exacerbating volunteer bias. Framing effects, defined as how researchers advertise or describe their study (Anderson et al., 2022), could potentially produce volunteer bias in an online recruitment environment. Indeed, August et al. (2018) showed that titles framed as “learning about oneself” attracted participants motivated to participate due to boredom, which in turn was associated with lower attentiveness and higher dropout. Thus, framing effects may make online studies especially prone to volunteer bias.

One area that frequently uses online self-report surveys and where volunteer bias might arise is relationship research, which examines experiences in singlehood and romantic relationships. A small body of research shows that studies and programs advertised for couples tend to attract a particular type of couple that differs from non-participants across demographic characteristics and well-being outcomes such as commitment levels. For example, higher education and income level predict higher response to mailed recruitment flyers (Karney et al., 1995). Longitudinal research further shows that romantic dyads who participate in studies together report greater individual and relationship well-being (Barton et al., 2020) and are more likely to stay together several years later compared with partnered individuals who participate alone (Park et al., 2021). Similarly, among engaged couples, the partner reporting higher commitment is more likely to express interest in marital education programs (Blair and Córdova, 2009). In general, women are more likely than men to express interest in relationship studies (Blair & Córdova, 2009), including longitudinal relationship studies (Hill et al., 1979). Volunteer bias also extends to relationship satisfaction; romantic partners of hospital patients who agreed to participate in a study reported lower negative affect and higher relationship satisfaction (Hagedoorn and colleagues, 2015). It is plausible that higher relationship satisfaction increases desires to self-disclose about your romantic relationship, including through research studies. This aligns with experience-sampling evidence that people share more relationship-relevant information on social media when they felt greater relationship satisfaction (Saslow et al., 2013). Overall, relationship research suggests volunteers may report higher well-being in outcomes like commitment, relationship quality and differ in demographics like gender. Notably, existing work involves recruitment into relatively intensive studies that are in-person, longitudinal, or dyadic, leaving open whether similar biases appear in less intensive mediums recruiting individual partnered participants.

With the recent rise in research on individuals who are *not* in romantic relationships, questions have emerged about who chooses to participate in singlehood studies and whether they represent the broader population. Theorizing around singlehood has posited that singles are a stigmatized group (DePaulo & Morris, 2005). Some singles may feel strongly about their singlehood identity, such as singlehood advocates who take pride in it, while others, especially involuntary singles, may feel more negatively towards their singlehood status. In both cases, feeling strongly about your singlehood identity (Fisher & Sakaluk, 2020), whether positively or negatively, could make singles more likely to participate in singlehood studies. Although volunteer bias might exist in singlehood research, evidence remains limited. One exception is Zillmann et al. (2014) who found that, amongst singles on a dating app, those who signed up for a study were older and lower in levels of education compared with singles who did not sign-up (Zillmann et al., 2014). Thus, volunteer bias may arise for singlehood studies, but this remains empirically unclear.

Prior research has identified volunteer bias in various areas of intensive relationship studies—such as longitudinal studies, dyadic research, and studies involving sensitive topics like sexuality. Given the widespread use of online recruitment, it is important to examine whether similar biases exist in online studies of singlehood and romantic relationships.

## Current Study

Given that one powerful mechanism through which volunteer bias can occur in online research is framing effects (Anderson et al., 2022; August et al., 2018), the current study examined framing effects in online singlehood and relationship studies across a host of commonly examined variables in the singlehood and romantic relationship domain, including demographics (i.e., age, gender, length of relationship status, education, socioeconomic status, employment, parental status), individual differences (i.e., extraversion, anxious and avoidant attachment), feelings about one’s current and alternative relationship status (i.e., perceived discrimination against singles, desire for a partner, desire for children, identification with and commitment to one’s relationship status), and well-being outcomes (i.e., life satisfaction, relationship status satisfaction, sexual satisfaction, loneliness, stress, autonomy, positive attitudes toward solitude). Measures were not selected systematically per se but rather were informed by a recent literature review (Girme et al., 2023) and guided by considerations of theoretical relevance, study length, and cost. For example, previous work showed that, amongst the five-factor personality traits, extraversion was the strongest predictor of being partnered (Hoan & MacDonald, 2024; Stern et al., 2024) and may influence who opts into relationship-focused studies. Similarly, autonomy was chosen given its prevalence in qualitative accounts of singlehood experiences (Dennett et al., 2025) and may shape interest in studies framed around singlehood.

Our research examined outcomes across three separate study posting conditions, each with a different framing in its title and description: a study on “people’s lifestyles,” a study on “singlehood and people’s lifestyles,” and a study on “romantic relationships and people’s lifestyles.” This design aimed to determine whether these different framings would select for single and partnered individuals that differed across the measured outcomes. While our primary focus was on testing the comparability of the samples’ characteristics across postings, we also conducted additional analyses, addressing the question of generalizability in effects. It is possible that rather than or in addition to affecting the mean levels of characteristics, certain relationships between variables manifest differently depending on the framing condition. To this end, we focused on the well-established links between individual differences—extraversion, attachment anxiety, and attachment avoidance—and well-being (Costa & McCrae, 1980; Karreman & Vingerhoets, 2012) and examined if these associations varied across conditions^1^.

## Study 1

### Method

#### Ethics and Open Practices Statement

Participant recruitment and procedures for this study were approved by the Research Ethics Board at the University of Toronto (Protocol #: 45223). All the data, study materials, and code needed to reproduce the primary results in this paper can be found on the OSF repository at https://osf.io/vx6p3/. We report all measures, manipulations, and participants excluded across both studies. Importantly, none of the methodological or analytical procedures in this study were preregistered as this research was exploratory.

##### Participants

Participants were recruited through the online crowdsourcing website Prolific. The postings were made available only to participants whose relationship status aligned with the eligibility criteria such that only singles saw the singles study posting and only partnered individuals saw the relationship study posting while no filter was applied for the general condition postings. In our pre-screeners, we recruited participants who were between the ages of 20 and 59 and participants who had been single or partnered for at least 6 months. Our power analysis determined a minimum sample size of 1,548 for a desired power of 95%, an alpha criterion of 5%, and a small to medium Cohen’s *f* effect size of 0.10. As we did not have existing pilot data or relevant literature on which to draw, our estimated effect size was based on existing research examining framing effects in sexuality studies (Dawson et al., 2019; Wiederman, 1999).

A total of 1,607 participants completed the study. We excluded participants who did not consent to the study (*n* = 8), failed both of our attention checks (*n* = 3), reported that they were dishonest (*n* = 5), reported that they did not want their data used (*n* = 2), as well as participants whose relationship length exceeded their age (*n* = 2). We also excluded participants who agreed to most items in a scale with reverse-scored statements, as this suggests poor attention paid to the study (Hinz et al., 2007). To do this, we created acquiescence scores by aggregating participants’ scores across the extraversion scale, which includes reverse-scored items for half of the statements, without reverse scoring any items. We then excluded participants who responded highly on both positive and negatively worded items by removing participants (*n* = 4) with scores beyond 3 standard deviations from the mean, based on visual inspection of the plotted acquiescence scores. After applying these exclusions, 1,595 participants were included in the analyses^2^.

Single participants (*n* = 822) were 27.15 years old on average with an average singlehood length of 61.76 months (*SD* = 93.01). Of these participants, 392 identified as men, 409 identified as women, and 19 identified as nonbinary. Most participants identified as heterosexual (*n* = 568), bisexual (*n* = 80), or homosexual (*n* = 42). Participants reported having a Bachelor’s degree (*n* = 299), a high school degree (*n* = 171), some college education (*n* = 166), while the remaining participants reported having a graduate degree (*n* = 112) or less than high school education (*n* = 5).

Partnered participants (*n* = 775) were 29.75 years old on average with an average relationship length of 64.57 months (*SD* = 69.75). Among the partnered participants, 387 identified as men, 364 identified as women, and 24 identified as non-binary. Participants mostly identified as Heterosexual (*n* = 687), Bisexual (*n* = 70), or Homosexual (*n* = 36). Participants reported having a bachelor’s degree (*n* = 344), a high school degree (*n* = 149), some college education (*n* = 138), a graduate degree (*n* = 194), or less than a high school degree (*n* = 9). Partnered participants were primarily from Europe (*n* = 472), South Africa (*n* = 177), Americas (*n* = 98), Asia (*n* = 36), Australia and New Zealand (*n* = 9), or did not report (*n* = 17; see Supplementary Table 1a for full participant characteristics for each study condition).

#### Procedure

Our study was a three-factor design: general condition, singlehood condition, relationship condition. Across these conditions, we varied three different study *titles* to advertise the research (one for each condition), while holding the study *description* constant across each condition (see Table 1). In the general condition, we used the generic title, “Study on People’s Lifestyles” to recruit both single and partnered individuals. The single and partnered conditions were titled explicitly as studies about singles (“Study on Singlehood and People’s Lifestyles”) or romantic relationships (“Study on Romantic Relationships and People’s Lifestyles”). We further standardized consent forms such that the mention of “singlehood” or “relationships” was removed and only appeared in the study title and description during study selection. We staggered the posting times of our three study conditions rather than releasing them simultaneously to serve two important purposes: first, this avoided having participants make direct choices between the general, singlehood, and romantic relationship conditions which we felt better represented the circumstances under which participants would usually be making study participation decisions (e.g., participate in a singlehood study or not); second, presenting each condition at different times prevents a bias towards participants from specific regions of the world being more likely to participate in a particular condition because of the time at which it was available. As such, we posted each study on different times each day in Eastern Standard Time (see Table 2). Over the span of 6 days, we posted one of three conditions in the morning (9:00 a.m. ET), afternoon (2:00 p.m. ET), and evening (7:00 p.m. ET), alternating the conditions for each time to avoid order effects^3^. For example, on Sunday, we collected a subset of the singles condition in the morning, a subset of the relationship condition in the afternoon, and a subset of the general condition in the evening. On Monday, we rotated the conditions to a different time. This rotation continued until all possible posting orders were exhausted. We followed this procedure so as to not introduce confounds with the date and time of recruitment (Ryan et al., 2010). Importantly, postings across all conditions were made available only to participants who had not previously participated in any previous condition and given that all postings were staggered such that only one was active at a time, this removed the possibility that participants could see or complete more than one condition. Moreover, the same pay rate of £9 per hour was used across all study conditions so that results were not confounded by differences in pay. The study was estimated and advertised to take approximately 11 minutes for participants in all conditions, translating to £1.65 upon study completion. All participants were pre-screened on their relationship status at the start of the survey to determine eligibility and, for the general condition, to ensure a balanced sample of single and partnered individuals.

**Table 1.** Study Descriptions.

Condition	Title	Description
General Condition	Study on People’s Lifestyles	This study will ask you about your demographic characteristics and life experiences.
Singles Condition	Study on Singlehood and People’s Lifestyles	
Relationship Condition	Study on Romantic Relationships and People’s Lifestyles	

**Table 2.** Posting Schedule of Study Conditions.

Day of the week	Time of posting		
	Morning (9am ET)	Afternoon (2pm ET)	Evening (7pm ET)
Sunday	Single Condition	Relationship Condition	General Condition
Monday	Single Condition	General Condition	Relationship Condition
Tuesday	Relationship Condition	Single Condition	General Condition
Wednesday	Relationship Condition	General Condition	Single Condition
Thursday	General Condition	Single Condition	Relationship Condition
Friday	General Condition	Relationship Condition	Single Condition

### Measures

The following measures were administered across the three conditions, although some measures were only applicable to, and thus administered to, single or partnered participants exclusively. See Table 3 for full details on which measures were administered to participants depending on their relationship status (see Table 2a and Table 2b in the Supplementary Materials for a correlation table of all primary variables).

**Table 3.** Measures Administered Based on Relationship Status.

Measure	All participants	Single	Partnered
Extraversion	X		
Attachment Style	X		
Perceived Discrimination Against Singles	X		
Desire for a Partner		X	
Voluntary Singlehood		X	
Identification with In-Group Status	X		
Commitment to Singlehood		X	
Life Satisfaction	X		
Satisfaction with Relationship Status	X		
Sexual Satisfaction	X		
Loneliness	X		
Stress	X		
Autonomy	X		
Positive Solitude Scale	X		
Investment Model Scale			X
Desire for Children	X		

*Note.* Scales were administered based on relationship status irrespective of study condition.

#### Demographics

##### Age

Age was measured as age in years.

##### Relationship Status Length

Relationship status length, or the length of time in which participants were single or partnered, was measured by asking participants to report how long they were single or in a relationship in months.

##### Socioeconomic Status

Socioeconomic status was measured using the MacArthur Scale of Subjective Social Status (Adler et al., 2000). This measure asks participants to place themselves on a 10-step ladder where the lowest rung represents “the people who are the worst off” while the top of the ladder represents “the people who are the best off” in terms of their money, education, and jobs.

##### Education

Education was measured by asking participants what the highest degree or level of school they have completed and if they were currently enrolled as students, what the highest degree they received was. Participants were shown the following options: less than high school degree, high school degree or equivalent (e.g., GED), some college but no degree, bachelor’s degree, or graduate degree.

##### Employment Status

Employment status was measured by asking participants to select the option that best describes their current employment status including being employed full-time, employed part-time, being out of work and/or currently looking for work, a homemaker, a student, or retired.

##### Parental Status

Parental status was measured by asking participants how many children they had, and if they had none, they were instructed to report “0.”

#### Individual Differences

##### Extraversion

Extraversion was measured using the 12-item extraversion subscale from the Big Five Inventory–2 (BFI-2; Soto & John, 2017; αs > 0.84; for example, “Is outgoing, sociable”). Items were rated on a 5-point scale ranging from 1 (*disagree strongly*) to 5 (*agree strongly*).

##### Attachment

Attachment styles were measured using the Experiences in Close Relationship Scale (ECR)–short form (Wei et al., 2007). The scale is composed of six items measuring attachment avoidance (αs > 0.61; e.g., “I try to avoid getting too close to others”) and six items measuring attachment anxiety (αs > 0.77; e.g., “I need a lot of reassurance that I am loved by others”). Items were rated on a 7-point scale ranging from 1 (*strongly disagree*) to 7 (*strongly agree*). Given the relatively low reliability observed in the alpha coefficients of the attachment avoidance subscale, we conducted Confirmatory Factor Analyses (CFA) separately for single and partnered individuals to examine the factor structure of this six-item subscale (see full details in the Supplemental Materials Appendix D). This CFA revealed that a reduced subscale demonstrated improved model fit, but that results did not vary after using a revised composite of attachment avoidance. Thus, the results reported below use the full items from the original subscale.

#### Feelings About One’s Current and Alternative Relationship Status

##### Perceived Discrimination Against Singles

Perception of stigma toward singles was measured using two items from Fisher and Sakaluk (2020), namely, “To what extent do you think that people who are single experience discrimination?” and “To what extent have you personally experienced discrimination for when you were single?” (αs > 0.87). Both items were rated on a 7-point scale ranging from 1 (*not at all*) to 7 (*a great deal)*.

##### Desire for a Partner

Desire for a partner was measured using a five-item scale developed in our lab to capture individual differences in how much a person wants a romantic partner (α_single_ = 0.93; e.g., “I want to have a romantic partner”). The validity of this scale is supported by evidence that individuals higher in desire for a partner are more likely to transition from singlehood to partnership (MacDonald et al., 2025). Items were rated on 7-point scales ranging from 1 (*strongly disagree*) to 7 (*strongly agree*).

##### Voluntary Singlehood

Voluntary singlehood, or the extent which individuals choose to be single, was measured using a one-item scale from Hostetler (2009) that states “It is my choice to be single.” This item was rated on a 5-point scale ranging from 1 (*strongly disagree*) to 5 (*strongly agree*).

##### Identification With In-Group Status

Identification with one’s in-group status, either as a single or partnered person, was measured using measures of in-group identification from Leach and colleagues (2008). This measure was adapted such that participants were asked about their identity as a single or a person in a romantic relationship depending on their reported relationship status. This measure is composed of subscales capturing one’s in-group solidarity (α_single_ = 0.87; α_partnered_ = 0.90; e.g., “I feel a bond with [singles/people in romantic relationships]”), satisfaction with their identity (α_single_ = 0.90; α_partnered_ = 0.89; e.g., “I am glad to be [single/a person in a romantic relationship]”), centrality (α_single_ = 0.70; α_partnered_ = 0.84; e.g., “The fact that I am [single/a person in romantic relationship] is an important part of my identity”), self-stereotyping (α_single_ = 0.90; α_partnered_ = 0.91; e.g., “I am similar to the average [single/a person in romantic relationship]”), and homogeneity (α_single_ = 0.91; α_partnered_ = 0.90; e.g., “[Singles/People in romantic relationships] are very similar to each other”). All items were rated on a 7-point scale ranging from 1 (*strongly disagree*) to 7 (*strongly agree*).

##### Commitment to Singlehood

Commitment to singlehood was measured using the four-item subscale of commitment from the Investment Model Scale for Singles (IMS-S; Beauparlant et al., 2024; α_single_ = 0.91; e.g., “I am committed to my current single life”). Items were rated on a 7-point scale ranging from 1 (*strongly disagree*) to 7 (*strongly agree*).

##### Desire for Children

Desire for children was measured using five items assessing the number of children they would like to have (Testa et al., 2016), their desire for a future child (Miller, 2011; rated on a scale of 0 = *no desire* to 10 = *very strong desire*), as well as their likelihood of having a child in the future (Household, Income and Labour Dynamics in Australia, 2001; rated on a scale of 0 = *definitely no* to 10 = *definitely yes*; αs > 0.91).

#### Well-Being

##### Life Satisfaction

Life satisfaction was measured using the five-item Satisfaction with Life Scale (Diener et al., 1985; α_single_ = 0.89; α_partnered_ = 0.90; e.g., “I am satisfied with my life”). Items were rated on a 7-point scale ranging from 1 (*strongly disagree*) to 7 (*strongly agree*).

##### Satisfaction With Relationship Status

Satisfaction with relationship status was measured using the four-item Satisfaction with Relationship Status Scale (Lehmann et al., 2015; α_single_ = 0.90; α_partnered_ = 0.92; e.g., “How happy are you with your current status?”). Participants were asked to think about their current status as referring to their single or partnered relationship status. Items were rated on a 4-point scale ranging from 1 (*not at all*) to 4 (*to a great extent*).

##### Sexual Satisfaction

Sexual satisfaction was measured using the four-item Satisfaction with Sex Life Scale–Revised (Park & MacDonald, 2022; α_single_ = 0.96; α_partnered_ = 0.98; e.g., “I am satisfied with my sexual life”). Participants were asked to think about how well each statement reflected their thoughts and feelings about their sexual life, including sexual activities they may or may not be engaging in. Items were rated on a 7-point scale ranging from 1 (*not at all*) to 7 (*extremely*).

##### Loneliness

Loneliness was measured using the 10-item UCLA Loneliness Scale (Russell, 1996; α_single_ = 0.89; α_partnered_ = 0.90; e.g., “I feel completely alone”). Items were rated on a 10-point scale ranging from 0 (*I never feel this way*) to 3 (*I often feel this way*).

##### Stress

Stress was measured using the 10-item Perceived Stress Scale (Cohen et al., 1994; α_single_ = 0.84; α_partnered_ = 0.84). Although the original scale asks participants to think about their stress in the last month, we adapted the scale to measure tendencies to feel stress by removing that component and changing the phrasing to refer to their general dispositions (e.g., “How often do you feel upset because of something that happened unexpectedly?”). Items were rated on a scale of 0 (*never*) to 4 (*very often*).

##### Autonomy

Autonomy, or the extent to which one feels free to do what they want, was measured using three items devised by our lab. Namely, participants were asked to rate the following three questions on a scale of 0 (*never*) to 4 (*very frequently*): “How often do you engage in each of the following activities?: Taking advantage of. . .” 1) my freedom to do what I want to do, 2) my independence, 3) being able to make choices for myself (α_single_ = 0.90; α_partnered_ = 0.91).

##### Positive Solitude

Positive solitude, or the degree to which people enjoy their alone time, was measured using the nine-item Positive Solitude Scale (Palgi et al., 2021; α_single_ = 0.89; α_partnered_ = 0.90; e.g., “When I am by myself, I can achieve the high level of focus that I need”). Items were rated on a scale of 1 (*not at all*) to 5 (*most of the time*).

#### Investment Model Scale

Predictors of persistence in one’s romantic relationship, including relationship satisfaction (α_partnered_ = 0.96; e.g., “My relationship is close to ideal”), quality of alternatives (α_partnered_ = 0.79; e.g., “The people other than my partner with whom I might become involved are very appealing”), investment size (α_partnered_ = 0.76; e.g., “I feel very involved in our relationship—like I have put a great deal into it”), and commitment (α_partnered_ = 0.79; e.g., “I am committed to maintaining my relationship with my partner”), were measured using 13 items from the Investment Model Scale–Shorter Version (IMS-S; Rodrigues & Lopes, 2013). Items were rated on a scale of 0 (*do not agree at all*) to 8 (*agree completely*).

### Results

#### Single Participants: Differences Between Singles Condition and General Condition

##### Mean Sample Characteristics Across Framing Conditions

To examine differences between single participants in the “Singlehood and People’s Lifestyles” condition and the general “People’s Lifestyles” condition, we conducted a series of t-tests (for continous outcomes) as well as Chi-squared tests (for categorical outcomes) and applied the Benjamini–Hochberg (BH) procedure to control for the false discovery rate (FDR) and account for family-wise error accumulation from multiple tests (Table 4). The FDR, in contrast to the Bonferroni–Holm Correction method (Holm, 1979), controls for Type I errors rates while allowing for greater power than the Bonferroni–Holm Correction which sacrifices power for reduction of Type I errors, but consequently, may produce higher rates of Type II errors (Thissen et al., 2002). As such, results are reported and interpreted in light of BH corrections.

**Table 4.** T-Test Results and Descriptives for the Singles Condition and General Condition Singles Across All Variables of Interest.

Variables	Singles only condition		General condition: singles		*t*	*χ²*	*p*	*α*_adj_	*d*	*V*	95% CI
	M	*SD*	M	*SD*							
Demographics											
Age	27.26	7.19	27.11	7.22	0.28	—	.78	.04	0.02	—	[-0.89, 1.18]
Singlehood Length	68.07	99.67	56.08	86.90	1.76	—	.07	.08	0.13	—	[-1.35, 25.31]
Socioeconomic Status	5.25	1.57	5.39	1.50	-1.22	—	.223	.01	-0.09	—	[-0.36, 0.08]
Education	3.43	1.01	3.45	1.07	—	7.72	.112	.04	—	0.10	—
Employment Status	2.75	1.67	2.81	1.64	—	1.61	.830	.03	—	0.05	—
Parental Status	0.10	0.36	0.13	0.51	—	5.49	.139	.02	—	0.09	—
Individual Differences											
Extraversion	2.92	0.72	2.90	0.70	0.31	—	.755	.04	0.02	—	[-0.09, 0.12]
Anxious Attachment	4.11	1.25	4.06	1.17	0.63	—	.531	.03	0.05	—	[-0.12, 0.23]
Avoidant Attachment	3.81	1.03	3.79	0.96	0.35	—	.730	.04	0.03	—	[-0.12, 0.17]
Feelings about Relationship Status											
Perceived Discrimination	2.86	1.67	2.73	1.60	1.09	—	.276	.02	0.08	—	[-0.10, 0.36]
Desire for a Partner	4.45	1.70	4.55	1.57	-0.80	—	.425	.03	-0.06	—	[-0.33, 0.14]
Voluntary Singlehood	3.44	1.25	3.34	1.26	1.06	—	.290	.02	0.08	—	[-0.08, 0.28]
Identification with In-Group Status	3.61	0.98	3.62	0.96	-0.11	—	.912	.05	0.01	—	[-0.15, 0.13]
Commitment to Singlehood	3.30	1.64	3.17	1.65	1.06	—	.288	.02	0.08	—	[-0.11, 0.36]
Desire for Children	2.42	2.35	2.43	2.21	-0.05	—	.960	.05	0.00	—	[-0.33, 0.32]
Well-Being											
Life Satisfaction	3.45	1.43	3.33	1.32	1.19	—	.234	.01	0.09	—	[-0.08, 0.32]
Relationship Status Satisfaction	2.34	0.82	2.30	0.80	0.71	—	.480	.03	0.05	—	[-0.07, 0.16]
Sexual Satisfaction	2.78	1.73	2.56	1.63	1.75	—	.081	.01	0.13	—	[-0.03, 0.45]
Loneliness	1.33	0.73	1.33	0.73	0.09	—	.926	.05	0.01	—	[-0.10, 0.11]
Perceived Stress	2.16	0.66	2.10	0.60	1.12	—	.261	.01	0.08	—	[-0.04, 0.14]
Autonomy	2.93	0.91	2.73	0.90	**3.00**	—	**.003**	**.002**	0.22	—	[0.07, 0.33]
Positive Solitude	3.77	0.80	3.80	0.71	-0.59	—	.553	.03	-0.04	—	[-0.14, 0.08]

*Note. M* = Mean; *SD* = Standard Deviation; *α*_adj_ = adjusted critical values after applying the Benjamini-Hochberg method; *d* = Cohen’s D effect size; *V* = Cramer’s V effect size. Note that Chi-squared (*χ²*) and Cramer’s V statistics only applied to categorical outcomes; as such, “—” indicates no applicable value. Bolded values indicate statistically significant coefficients at *p* < .05.

Overall, when examining differences in singles’ demographic characteristics across the singles condition and general condition, we found no differences including for age, singlehood length, socioeconomic status, education, employment status, and parental status. Likewise, no differences across individual trait differences were found including in extraversion, anxious attachment, avoidant attachment, or with outcomes related to feelings about one’s current and alternative relationship status, including perceived discrimination against singles, desire for a partner, voluntary singlehood, identification with in-group status, commitment to singlehood, as well as desire for children.

While singles did not differ across most well-being outcomes, including life satisfaction, relationship status satisfaction, sexual satisfaction, loneliness, perceived stress, or perceptions of positive solitude, singles across conditions did demonstrate significant differences in their levels of autonomy, *t*(745) = 3.00, *p* = .003, such that participants in the singles study condition reported higher autonomy than the general singles condition. However, after applying a FDR, this finding was no longer significant at α = 0.002 across 22 *t* values. Altogether, singles showed no significant differences between the “Singlehood and People’s Lifestyles” condition and the “People’s Lifestyles” condition across demographics, individual differences, feelings about one’s current and alternate relationship status, and well-being outcomes.

##### Moderation by Framing Condition

We ran multivariate regressions to test whether the associations between these individual differences and well-being outcomes varied by framing condition (see Supplementary Materials ‘Appendix F’ for full model results). Only two interactions emerged suggesting stronger associations in the general (vs. singles-specific) condition. Specifically, the negative association between extraversion and loneliness was stronger in the general condition (*b* = -0.50, *SE* = 0.05, *p* < .001) than in the singles-specific condition (*b* = -0.33, *SE* = 0.05, *p* < .001) and the positive association between attachment avoidance and autonomy was stronger in the general condition (*b* = 0.30, *SE* = 0.03, *p* < .001) than the singles-specific condition (*b* = 0.26, *SE* = 0.03, *p* < .001). No other moderations emerged for attachment anxiety or avoidance (full results in Supplementary Materials).

#### Partnered Participants: Differences Between Partnered Condition and General Condition

Mean Sample Characteristics Across Framing Conditions

To examine differences between partnered participants in the “Romantic Relationships and People’s Lifestyles” condition and the general “People’s Lifestyles” condition, we applied the same approach as with the singles and conducted a series of t-tests and Chi-squared tests (Table 5). We then applied the Benjamini–Hochberg method to account for the FDR from multiple tests.

**Table 5.** T-Test Results and Descriptives for Partnered Participants in the Relationship Condition and General Condition Across All Variables of Interest.

Variables	Relationship condition		General condition: partnered		*t*	*χ²*	*p*	*α*_adj_	*d*	*V*	95% CI
	M	*SD*	M	*SD*							
Demographics											
Age	29.67	7.99	29.83	8.41	-0.28	—	.78	.04	-0.02	—	[-1.29, 0.96]
Relationship Length	66.00	66.54	63.18	73.20	0.58	—	.56	.03	0.04	—	[-6.78, 12.41]
Socioeconomic Status	5.66	1.42	5.66	1.53	-0.03	—	.98	.05	0.00	—	[-0.21, 0.20]
Education	-0.18	1.07	3.69	1.04	-0.51	2.94	.57	.04	—	0.06	—
Employment Status	2.23	1.61	2.32	1.59	-0.77	5.51	.24	.02	—	0.08	—
Parental Status	0.50	0.83	0.43	0.86	1.08	7.83	.05	.02	—	0.10	—
Individual Differences											
Extraversion	3.23	0.65	3.12	0.73	2.14	—	.03	.004	0.15	—	[0.01, 0.20]
Anxious Attachment	3.88	1.18	3.89	1.26	-0.17	—	.87	.05	-0.01	—	[-0.18, 0.15]
Avoidant Attachment	3.38	0.92	3.45	1.02	-1.07	—	.28	.02	-0.08	—	[-0.21, 0.06]
Feelings about Relationship Status											
Perceived Discrimination	2.67	1.61	2.69	1.66	-0.24	—	.81	.05	-0.02	—	[-0.25, 0.20]
Identification with In-Group Status	4.78	1.05	4.70	1.08	1.13	—	.26	.01	0.08	—	[-0.06, 0.23]
Desire for Children	3.30	2.86	3.05	2.85	1.23	—	.22	.01	0.09	—	[-0.15, 0.64]
Well-Being											
Life Satisfaction	4.22	1.41	4.17	1.40	0.51	—	.61	.04	0.04	—	[-0.14, 0.24]
Relationship Status Satisfaction	3.22	0.72	3.26	0.74	-0.63	—	.53	.03	-0.04	—	[-0.13, 0.07]
Sexual Satisfaction	4.72	1.72	4.62	1.77	0.87	—	.39	.02	0.06	—	[-0.13, 0.35]
Loneliness	1.01	0.68	1.11	0.76	-1.99	—	.05	.01	-0.14	—	[-0.20, -0.001]
Perceived Stress	2.03	0.62	2.01	0.63	0.64	—	.53	.03	0.04	—	[-0.06, 0.11]
Autonomy	3.65	0.91	2.67	0.91	**15.41**	—	**< .001**	**.002**	1.08	—	[0.86, 1.11]
Positive Solitude	3.92	0.71	3.90	0.73	0.42	—	.68	.04	0.03	—	[-0.08, 0.12]
Investment Model Scale											
Relationship Satisfaction	6.28	1.78	6.14	1.93	1.05	—	.30	.02	0.07	—	[-0.12, 0.39]
Quality of Alternatives	3.52	2.05	3.40	2.25	0.77	—	.44	.03	0.05	—	[-0.18, 0.41]
Investment Size	5.53	1.69	5.43	1.92	0.74	—	.46	.03	0.05	—	[-0.15, 0.34]
Commitment	6.99	1.59	6.81	1.86	1.51	—	.13	.01	0.11	—	[-0.05, 0.42]

*Note. M* = Mean; *SD* = Standard Deviation; *α*_adj_ = adjusted critical values after applying the Benjamini-Hochberg method; *d* = Cohen’s D effect size; *V* = Cramer’s V effect size. Note that Chi-squared (*χ²*) and Cramer’s V statistics only applied to categorical outcomes; as such, “—” indicates no applicable value. Bolded values indicate statistically significant coefficients at p < .05.

When examining differences in partnered participants’ demographics, individual differences, and feelings about their current and alternative relationship status across conditions, we found no significant differences. This was true of all demographics including age, partnership length, socioeconomic status, education, employment status, and parental status. No differences were found in partnered participants’ individual trait differences as well, including in anxious attachment and avoidant attachment. Extraversion did not remain significant after applying FDR, *t*(805) = 2.14, *p* = .033 > BH Critical = 0.004. When examining differences across variables related to feelings about one’s current and alternative relationship status, we found no significant differences, including in perceived discrimination against singles, identification with in-group status, and desire for children.

Regarding differences in well-being and the relationship investment model, partnered individuals did not differ in their levels of life satisfaction, relationship status satisfaction, sexual satisfaction, loneliness, perceived stress, and perceptions of positive solitude across conditions. Notably, partnered participants differed in their autonomy across conditions, *t*(818) = 15.41, *p* < .0001 < BH Critical = 0.002, such that the relationship study condition reported higher autonomy than the general partnered condition. Overall, partnered participants did not differ in their well-being depending on the study framing, except for autonomy. Finally, when examining differences in partnered participants’ investment model outcomes, no differences emerged across conditions for relationship satisfaction, quality of alternatives, investment size, and commitment.

##### Moderation by Framing Condition

As with singles, we tested whether framing condition moderated associations between individual differences (extraversion, attachment anxiety, and attachment avoidance) and well-being in partnered participants. No significant interactions emerged for extraversion, indicating that its associations with the seven well-being outcomes were consistent across the general partnered and relationship study conditions. Framing condition did moderate the link between attachment anxiety and loneliness (*b* = -0.04, *SE* = 0.02, *p* = .01), with a stronger positive association in the general partnered condition (*b* = 0.30, *SE* = 0.02, *p* < .001) than in the relationship study condition (*b* = 0.21, *SE* = 0.02, *p* < .001). No other significant moderations were found, suggesting that, overall, insecure attachment–well-being links were similar across framing conditions.

##### Summary

Altogether, no differences were observed between partnered participants in the “Romantic Relationships and People’s Lifestyles” condition and the “People’s Lifestyles” condition with regards to their demographics, individual differences, feelings about one’s current and alternative relationship status, and most well-being outcomes except autonomy.

### Brief Discussion

The results of Study 1 provided almost no evidence of framing effects. Overall, individuals who participated in a study framed as a general lifestyle study did not differ meaningfully from those who participated in a study explicitly framed around their relationship status. A few exceptions emerged: singles in the singles condition reported higher autonomy, while partnered participants in the relationship study condition reported higher extraversion, lower loneliness, and greater autonomy. Furthermore, with the exception of three small significant interactions, the links between individual differences (extraversion, anxious attachment, avoidant attachment) and well-being did not vary by framing condition.

While results generally indicated little evidence for volunteer bias, a few variables showed potential effects. To confirm whether these few potential differences were weak signals of a real effect or random noise, we conducted a targeted replication, focusing only on measures that had shown small effect-size trends (*d* > 0.10). This approach would help us detect replicable effects without ballooning study costs or imposing unnecessary burden on participants by re-testing all variables. Single participants were assessed on relationship status length, sexual satisfaction, and autonomy, while partnered participants were assessed on extraversion, loneliness, and autonomy. Note that moderations by framing analyses were conducted per reviewer feedback, and as such, not all relevant variables were available in Study 2, preventing replication of the three interactions that emerged. As framing condition was almost completely unrelated to differential associations between individual differences and well-being, we suggest the few significant findings be interpreted cautiously as exploratory.

## Study 2

### Method

The procedures and measures used in Study 2 were the same as in Study 1, except that only measures for the above-mentioned outcomes were included.

#### Participants

Participants were recruited through Prolific. As with Study 1, we recruited participants who were 20 to 59 years old and who had been single or partnered for at least 6 months. Based on the results of Study 1, we conducted two power analyses, one for singles and one for partnered individuals for a desired power of 80% and an alpha criterion of 5%. For singles, based on a Cohen’s *d* of 0.13 from the smallest effect being tested (sexual satisfaction), our power analysis determined a minimum sample size of 733 per condition (total 1,466). For partnered individuals, based on a Cohen’s *d* of 0.14 from the smallest effect being tested (loneliness), our power analysis determined a minimum sample size of 632 per condition (total 1,264).

A total of 3,352 participants entered our study. We excluded participants who did not consent to the study (*n* = 4), failed attention checks (*n* = 8), whose relationship status length exceeded their age (*n* = 4), reported that they were single in the partnered conditions (*n* = 20) or partnered in the single conditions (*n* = 392), and who failed to complete the study (*n* = 330). As in Study 1, we excluded participants based on their levels of acquiescent responding. However, this was only conducted on partnered participants who completed the extraversion scale which includes reverse-scored items; meanwhile, single participants in Study 2 did not complete scales with reverse-scored items that would allow us to detect acquiescent responding. To calculate acquiescent scores, we used the same approach as described in Study 1 and excluded participants who responded highly on both positive and negatively worded items (*n* = 10). After applying all the exclusions described above, 2,777 participants were included in the analyses.

Single participants (*n* = 1,470) were 30.48 years old on average with an average singlehood length of 70.63 months (*SD* = 97.98). Of these participants, 715 identified as men, 717 identified as women, 36 identified as nonbinary, and 2 preferred not to disclose. Most participants identified as Heterosexual (*n* = 1,115), Bisexual (*n* = 138), or Homosexual (*n* = 74). Participants reported having a Bachelor’s degree (*n* = 593), a high school degree (*n* = 283), some college education (*n* = 322), while the remaining participants reported having a graduate degree (*n* = 244) or less than high school education (*n* = 28). Single participants were mostly from Europe (*n* = 736), the Americas (*n* = 397), and South Africa (*n* = 237), with smaller numbers from Asia (*n* = 59) and Australia/New Zealand (*n* = 37); four did not report.

Partnered participants (*n* = 1,304) were 31.88 years old on average with an average relationship length of 78.61 months (*SD* = 82.63). Among the partnered participants, 535 identified as men, 749 identified as women, and 22 identified as nonbinary, and 1 preferred not to disclose. Participants mostly identified as Heterosexual (*n* = 1,072), Bisexual (*n* = 109), or Homosexual (*n* = 55). Participants reported having a bachelor’s degree (*n* = 625), a high-school degree (*n* = 145), some college education (*n* = 197), a graduate degree (*n* = 329), or less than a high school degree (*n* = 11). Partnered participants were primarily from Europe (*n* = 518), South Africa/Zimbabwe (*n* = 487), the Americas (*n* = 190), Asia (*n* = 54), Australia/New Zealand (*n* = 49); six did not report (see Supplementary Materials Appendix B for full demographics).

#### Procedure

We followed the same procedures as in Study 1 such that we advertised our study using three different study titles for each condition (Table 1), alternating times of posting over the span of 6 days (see Table 2). Again, postings were made available only to participants who had not previously participated and the same pay rate of £9 per hour was used across all study conditions. The study completion time was 3 minutes for single participants (£0.45 for completion) and 5 minutes for partnered participants (£0.75 for completion). As such, to hold the advertised study time constant, we created two separate postings for the general condition based on relationship status: a 3-minute general lifestyle condition made visible only to single participants and a 5-minute general lifestyle condition made visible only to partnered participants. All participants were prescreened, and those with an ineligible relationship status were excluded from the start.

### Measures

#### Demographics

##### Relationship Status Length

Relationship status length was measured in the same way as in Study 1.

#### Personality

##### Extraversion

Extraversion was measured in the same way as in Study 1 (α_partnered_ = 0.86).

#### Well-Being

##### Loneliness

Loneliness was measured in the same way as in Study 1 (α_partnered_ = 0.91).

##### Sexual Satisfaction

Sexual satisfaction was measured in the same way as in Study 1

(α_single_ = 0.95).

##### Autonomy

Autonomy was measured in the same way as in Study 1 (α_single_ = 0.86; α_single_ = 0.88).

### Results

#### Single Participants: Differences Between Singles Condition and General Singles Condition

As in Study 1, to examine differences between single participants in the singles study condition and the general condition, we conducted a series of independent samples t-tests (see Table 6). Given that no categorical outcomes were examined in Study 2, no Chi-squared tests were used.

**Table 6.** T-Test Results and Descriptives for Between Conditions Across All Variables of Interest: Single Condition and General Condition.

Variables	Single condition		General condition: single		*t*	*p*	*Cohen’s d*	95% CI
	M	*SD*	M	*SD*				
Demographics								
Singlehood Length	74.16	101.06	67.06	94.71	1.39	0.165	0.07	[-2.93, 17.11]
Well-Being								
Sexual Satisfaction	3.01	1.80	3.02	1.73	-0.11	0.910	0.01	[-0.19, 0.17]
Autonomy	3.01	0.84	3.02	0.83	-0.11	0.914	0.01	[-0.09, 0.08]

We observed no significant differences between the singles condition and the general condition singles in how long they have been single for, *t*(1463) = 1.39, 95% *CI* = [−2.93, +17.11], *p* = .165. Likewise, singles did not show significant differences in their satisfaction with their sex life, *t*(1467) = −0.11, 95% *CI* = [−0.19, 0.17], *p* = .910, or the extent to which they feel autonomous, *t*(1468) = −0.11, 95% *CI* = [−0.09, +0.08], *p* = .914, across conditions.

#### Partnered Participants: Differences Between Partnered Condition and General Condition

As in Study 1, to examine differences between partnered participants in the relationship study condition and the general condition, we conducted a series of independent samples t-tests (see Table 7).

**Table 7.** T-Test Results and Descriptives for Between Conditions Across All Variables of Interest: Relationship Condition and General Condition.

Variables	Relationship condition		General condition: partnered		*t*	*p*	*Cohen’s d*	95% CI
	M	*SD*	M	*SD*				
Individual Differences								
Extraversion	3.23	0.73	3.23	0.73	-0.09	0.926	0.01	[-0.08, 0.08]
Well-Being								
Loneliness	1.04	0.71	1.03	0.67	0.21	0.837	0.01	[-0.07, 0.08]
Autonomy	2.86	0.87	2.89	0.84	-0.64	0.525	-0.04	[-0.12, 0.06]

We observed no significant differences between the partnered only condition and the general condition partnered participants in their trait extraversion, *t*(1304) = −0.09, 95% *CI* = [−0.08, +0.08], *p* = .926. Similarly, we observed no significant differences between the relationship condition and the general condition partnered participants in their loneliness, *t*(1304) = 0.21, 95% *CI* = [−0.07, +0.08], *p* = .837, or in the extent to which they felt autonomous, *t*(1305) = −0.64, 95% *CI* = [−0.12, +0.06], *p* = .525.

### Brief Discussion

In Study 2, we aimed to replicate the suggestive findings of Study 1 whereby we initially found minimal differences between status specific and general conditions. The results of Study 2 suggested that none of the Study 1 effects were replicable; there were no significant differences when comparing single individuals in the singles study condition versus singles in the general condition, nor were there differences when comparing partnered individuals in the relationship condition versus partnered participants in the general condition.

## General Discussion

Our results lend support to a lack of volunteer bias in singlehood and relationship studies conducted online via Prolific. Specifically, studies explicitly framed around singlehood or relationships do not appear to select for a biased sample of participants within this particular online pool. We found no replicable, significant framing effects across a wide variety of outcomes, including demographics, individual differences, feelings about one’s current and alternative relationship status, or well-being. Our findings provide no evidence that online recruitment methods—which are among the most common approaches in social psychology (Buhrmester et al., 2018)—generate volunteer bias or threaten validity in singlehood and romantic relationship research. This insight is particularly valuable for the emerging field of singlehood studies, where researchers are still establishing foundational knowledge, and often employ online samples (Beauparlant et al., 2024; Dupuis & Girme, 2024; MacDonald & Park, 2022). The results provide initial support for the validity of online recruitment methods as singlehood researchers build an initial knowledge base.

Our findings are in contrast to prior evidence of volunteer bias in dyadic relationship and sexuality research (Anderson et al., 2022; Barton et al., 2020; Park et al., 2021; Regan, 2008). A key difference may be the nature of online studies. Previous research has examined contexts like flyer recruitment (Karney et al., 1995), in-person couple interviews (Park et al., 2021), and relationship education programs (Blair & Córdova, 2009), where participants carefully weigh study interest against significant time and energy demands. By comparison, online studies require minimal commitment and often offer financial incentives, which could reduce selective participation based on study framing. For example, completing a brief online study about your romantic relationship demands less investment than a longitudinal, in-person study, so participants may pay less attention to titles or descriptions for brief online studies, reducing the impact of framing effects.

Furthermore, it is possible that our null findings can be explained by the available pool of participants on Prolific. While Prolific aims to maintain a diverse research pool, the demographics of Prolific participants are typically younger and more familiar with technology, English-speaking, and often possess some form of higher education (Turner et al., 2021). This might restrict the range of participants who could be selected into our study. For example, it is possible that effects might differ in populations such as older adults. Previous work has shown that younger singles tend to be less satisfied with singlehood, but singles 40 and older tend to show increasing levels of satisfaction with singlehood (Böger & Huxhold, 2018; Park et al., 2022). As such, older singles might show different levels of interest in participation in singlehood-framed studies that is affected by their greater average comfort with being single.

### Strengths, Limitations, and Future Directions

Our study possesses numerous strengths. For one, across both studies, we use large, highly powered samples which allows for more reliable conclusions. In a study such as this, the ability to be confident that a null effect is meaningful is crucial, and our power analyses suggest confidence in the conclusion that framing effects are not present. Additionally, to isolate the framing effect manipulation, we controlled for potential confounds by removing acquiescent scoring and holding constant the costs of the studies, the study posting time and date, as well as ensuring that participants could not see or participate in the posting of more than one condition. This helped reduce the potential bias of sampling only geographical regions whose working hours align with our study postings as well as maintaining independent groups for comparison.

However, our study also holds limitations. First, our primary goal was to test whether different framing conditions produce volunteer bias by comparing sample characteristics across recruitment strategies. Such comparisons address sample comparability but not the generalizability of effects—the relationships *between* variables that researchers typically study. Our moderation analyses addressed this in part by examining whether framing condition moderated well-established links between individual differences and well-being outcomes. Nonetheless, fully assessing the generalizability of effects would require large-scale studies across diverse methodological variations, as in the Many Labs projects (Ebersole et al., 2016; Klein et al., 2018).

Second, it is possible that our manipulation was too weak to produce an effect. To maintain control in our study, we only manipulated a select few words in the description and title. However, this could have resulted in participants agreeing to the study without closely reading the title or fully understanding the study subject. Also, our base description of the study was a framing focused on “people’s lifestyles,” which could have introduced its own form of volunteer bias that our study was not set up to detect. It is possible (but unlikely) that there is something about people’s lifestyles as a subject that selected out people who may be affected by a relationship status volunteer bias.

Although we did not find evidence of volunteer bias in the context of our relationship status studies, this does not rule out its presence in other outcomes or areas of social and personality psychology. Domains like moral and political psychology commonly employ online self-report methods and may attract participants motivated to participate for personal interests in a way that biases the sample and results, such as if they feel strongly about their political or ethical views. Additionally, our study did not include a fully exhaustive list of relevant measures. For example, we did not implement experiential sampling methods to capture daily social behaviors and moods, which have previously been examined within the relationship literature (Impett et al., 2005). As such, future work could begin to examine online volunteer bias in other social psychological areas. Furthermore, it is also possible that framing effects could have an impact on other outcomes that we did not capture. Although we aimed to capture a host of key outcomes that are commonly used in the relationship and singlehood space, there may be other unmeasured outcomes in which framing effects might have an impact. As such, future research could examine additional outcome variables, such as open-ended responses about individuals’ experiences with singlehood and romantic relationships.

Of course, the absence of framing effects based on study title does not necessarily rule out the presence of general volunteer bias. While participants did not appear to self-select based on whether the study was explicitly framed around their relationship status or more generally framed, it is possible that our participants, across all conditions, differ from non-participants, such as those who would not take part in any type of online study (e.g., Burnham et al., 2018; Walters et al., 2018) or those who may not find psychological research of this nature (i.e., studies involving self-disclosure) particularly interesting. That said, because framing effect is one of the more common mechanisms that could amplify volunteer bias, and one with practical implications for researchers, our findings offer some reassurance about the negligible impact of this factor in relationship research.

Overall, our study opens a conversation attending to the possibility of volunteer bias in online recruitment in relationship status research. Future studies can extend these findings to further examine (lack of) volunteer bias in singlehood and romantic relationship studies while utilizing different manipulations such as longer descriptions catered toward single (e.g., a study about freedom and autonomy in singlehood) or partnered experiences (e.g., study about passionate love in your romantic relationship), qualitative versus quantitative studies, and online versus in person research. For example, a number of singlehood studies employ in-person recruitment of participants through university campus boards, research assistant recruitment, or online recruitment through social media platforms (Apostolou et al., 2024; Dennett & Girme, 2024). Within these contexts, which arguably require greater effort to sign up, volunteer bias might arise such that only participants who are intrinsically motivated in the topic will participate. One approach could be testing for volunteer bias by posting visually similar flyers on campus that vary only in their study content descriptions.

## Conclusion

Concerns about validity have long been present in psychology (Krantz & Dalal, 2000; Reips, 2000) and have intensified in the wake of the replication crisis (Fabrigar et al., 2020). Participant recruitment, especially online, is a nearly ubiquitous methodology employed in social psychology, including in singlehood and romantic relationship research. Past research has shown that volunteer bias can emerge in dyadic romantic relationship research (Park et al., 2021), as well as in relationship and sexuality research (Dollinger & Leong, 1993; Hill et al., 1979), suggesting that volunteer bias could similarly emerge in singlehood and relationship studies. However, the results of our study showed no such bias. This evidence helps address ongoing concerns about the validity of convenience samples and is particularly relevant to singlehood research—a growing area within relationship science that is being founded on online recruitment methods. Overall, given the widespread use of online studies, evidence that framing effects have minimal impact on volunteer bias offers researchers greater confidence in relying on online recruitment methods.

## Supplemental Material

sj-docx-1-psp-10.1177_01461672251390020 – Supplemental material for Who Signs Up for Singlehood and Romantic Relationship Studies? Examining Volunteer Bias in Online Recruitment Supplemental material, sj-docx-1-psp-10.1177_01461672251390020 for Who Signs Up for Singlehood and Romantic Relationship Studies? Examining Volunteer Bias in Online Recruitment by Elaine Hoan, Yoobin Park and Geoff MacDonald in Personality and Social Psychology Bulletin

## Data Availability

Data and code for the primary analyses are available at the Open Science Framework (https://osf.io/vx6p3/).

## Notes

1. We also examined whether the magnitude of the differences between single and partnered individuals depended on the posting conditions. That is, do single and partnered individuals appear more or less different when recruited under general vs. status specific framing? Only one moderation effect was observed in Study 1, and it was not replicated in Study 2. Full results are reported in the Supplemental Materials Appendix E.

2. Note that some participants met more than one of the exclusion criteria hence the total number of excluded participants not equating the number of exclusions in each exclusion criterion

3. Note that the presentation of measures within surveys were not randomized which may have introduced potential order effects.
