# Supplementary material for: Who Signs Up for Singlehood and Romantic Relationship Studies? Examining Volunteer Bias in Online Recruitment
Source: Pers Soc Psychol Bull. 2025 Dec 16;52(12):4066–81. doi: 10.1177/01461672251390020 (PMC13736306; doi:10.1177/01461672251390020)
Supplement: Supplementary file 1 [file sj-docx-1-psp-10.1177_01461672251390020.docx]

**Supplemental Materials**

Table of Contents

[Appendix A: Supplemental Table 1a. Study 1 Participant demographics by relationship status and study condition	2](#_Toc199251676)

[Appendix B: Supplementary Table 1b. Study 2 Participant demographics by relationship status and study condition	3](#_Toc199251677)

[Appendix C: Correlation Table of Main Variables by Relationship Status	4](#_Toc199251678)

[Appendix D: Supplemental Analyses: Confirmatory Factor Analysis with Avoidant Attachment	6](#_Toc199251679)

| **Appendix A: Supplemental Table 1a. Study 1 Participant demographics by relationship status and study condition** | | | |  |
| --- | --- | --- | --- | --- |
|  | **Condition** | | | |
| **Demographics** | **General (Single)** | **Single** | **General (Partnered)** | **Partnered** |
| **Age** | *M* = 27.04 (*SD* = 7.18) | *M* = 27.26 (*SD* = 7.19) | *M* = 29.82 (*SD* = 8.36) | *M* = 29.67 (*SD* = 7.99) |
| **Gender** | Man = 195 Woman = 219 Non-binary = 6 | Man = 197 Woman = 190 Non-binary = 13 | Man = 204 Woman = 144 Non-binary = 13 | Man = 183 Woman = 220 Non-binary = 11 |
| **Relationship Status Length** | *M =* 55.44 (*SD* = 86.34) | *M =* 68.07 (*SD* = 99.67) | *M =* 63.13 (*SD* = 72.96) | *M =* 66 (*SD* = 66.54) |
| **Sexual Orientation** | Heterosexual = 271 Homosexual = 22 Bisexual = 29 Queer = 7 Questioning = 9 Pansexual = 12 Asexual = 5 | Heterosexual = 297 Homosexual = 20 Bisexual = 51 Queer = 7 Questioni8ng =  Pansexual 2=  Asexual = 10 | Heterosexual = 352 Homosexual = 18 Bisexual = 36 Queer = 1 Questioning = 1 Pansexual = 7 Asexual = 1 | Heterosexual = 335 Homosexual = 18 Bisexual = 34 Queer = 8 Questioning = 1  Pansexual = 11 Asexual = 4 |
| **Socioeconomic Status** | *M =* 5.38 (*SD* = 1.50) | *M =* 5.25 (*SD* = 1.57) | *M =* 5.66 (*SD* = 1.56) | *M =* 5.66 (*SD* = 1.42) |
| **Education** | Less than Highschool = 5 Highschool = 91 Some college = 70 Bachelor degree = 135 Graduate degree = 60 | Less than Highschool = 8 Highschool = 80 Some college = 96 Bachelor degree = 164 Graduate degree = 52 | Less than Highschool = 5 Highschool = 67 Some college = 76 Bachelor degree = 174 Graduate degree = 98 | Less than Highschool = 4 Highschool = 82 Some college = 62 Bachelor degree = 170 Graduate degree = 96 |
| **Employment Status** | Full-time = 121 Part-time = 59 Out of work = 67  Homemaker = 3 Student = 111 Retired = 0 | Full-time = 147 Part-time = 57 Out of work =70  Homemaker = 4 Student = 120 Retired = 2 | Full-time = 208 Part-time = 63 Out of work = 50  Homemaker = 13 Student = 85 Retired = 1 | Full-time = 220 Part-time = 64 Out of work = 36  Homemaker = 6 Student = 85 Retired = 3 |

| **Appendix B: Supplementary Table 1b. Study 2 Participant demographics by relationship status and study condition** | | | |  |
| --- | --- | --- | --- | --- |
|  | **Condition** | | | |
| **Demographics** | **General (Single)** | **Single** | **General (Partnered)** | **Partnered** |
| **Age** | *M* = 30.58 (*SD* = 9.84) | *M* = 30.38 (*SD* = 9.23) | *M* = 31.62 (*SD* = 8.79) | *M* = 32.13 (*SD* = 8.97) |
| **Gender** | Man = 352 Woman = 366 Non-binary = 12 | Man = 363 Woman = 351 Non-binary = 22 | Man = 270 Woman = 360 Non-binary = 11 | Man = 265 Woman = 389 Non-binary = 9 |
| **Relationship Status Length** | *M =* 67.06 (*SD* = 94.71) | *M =* 74.16 (*SD* = 101.06) | *M =* 77.76 (*SD* = 82.31) | *M =* 79.43 (*SD* = 82.99) |
| **Sexual Orientation** | Heterosexual = 552 Homosexual = 35 Bisexual = 75 Queer = 11 Questioning = 15 Pansexual = 15 Asexual = 16 | Heterosexual = 563 Homosexual = 39 Bisexual = 63 Queer = 18 Questioning = 13 Pansexual = 13 Asexual = 14 | Heterosexual = 519 Homosexual = 27 Bisexual = 59 Queer = 6 Questioning = 9 Pansexual = 10 Asexual = 6 | Heterosexual = 553 Homosexual = 28 Bisexual = 50 Queer = 6 Questioning = 6  Pansexual = 10 Asexual = 6 |
| **Socioeconomic Status** | *M =* 5.26 (*SD* = 1.67) | *M =* 5.34 (*SD* = 1.66) | *M =* 5.86 (*SD* = 1.50) | *M =* 6.08 (*SD* = 1.49) |
| **Education** | Less than Highschool = 10 Highschool = 140 Some college = 161 Bachelor degree = 300 Graduate degree = 121 | Less than Highschool = 18 Highschool = 143 Some college = 161 Bachelor degree = 293 Graduate degree = 123 | Less than Highschool = 3 Highschool = 75 Some college = 95 Bachelor degree = 304 Graduate degree = 165 | Less than Highschool = 8 Highschool = 70 Some college = 102 Bachelor degree = 321 Graduate degree = 164 |
| **Employment Status** | Full-time = 323 Part-time = 113 Out of work = 123  Homemaker = 9 Student = 158 Retired = 6 | Full-time = 310 Part-time = 144 Out of work = 125  Homemaker = 6 Student = 151 Retired = 2 | Full-time = 394 Part-time = 81 Out of work = 56  Homemaker = 15 Student = 89 Retired = 6 | Full-time = 425 Part-time = 93 Out of work = 46  Homemaker = 19 Student = 79 Retired = 3 |

# **Appendix C: Correlation Table of Main Variables by Relationship Status**

**Table 2a. Correlation Tables of Primary Variables for Single Individuals Only**

| **Variables** | **1** | **2** | **3** | **4** | **5** | **6** | **7** | **8** | **9** | **10** | **11** | **12** | **13** | **14** | **15** | **16** | **17** | **18** | **19** |
| --- | --- | --- | --- | --- | --- | --- | --- | --- | --- | --- | --- | --- | --- | --- | --- | --- | --- | --- | --- |
| **1. Age** | — |  |  |  |  |  |  |  |  |  |  |  |  |  |  |  |  |  |  |
| **2. Extraversion** | -.01 | — |  |  |  |  |  |  |  |  |  |  |  |  |  |  |  |  |  |
| **3. Anxious Attachment** | -.14 | -.06 | — |  |  |  |  |  |  |  |  |  |  |  |  |  |  |  |  |
| **4. Avoidant Attachment** | .04 | -.46 | -.06 | — |  |  |  |  |  |  |  |  |  |  |  |  |  |  |  |
| **5. Life Satisfaction** | -.01 | .34 | -.21 | -.30 | — |  |  |  |  |  |  |  |  |  |  |  |  |  |  |
| **6. Status Satisfaction** | .08 | .08 | -.27 | .03 | .31 | — |  |  |  |  |  |  |  |  |  |  |  |  |  |
| **7. Sexual Satisfaction** | -.02 | .11 | -.06 | -.12 | .30 | .48 | — |  |  |  |  |  |  |  |  |  |  |  |  |
| **8. Stress** | -.10 | -.35 | .48 | .27 | -.52 | -.27 | -.16 | — |  |  |  |  |  |  |  |  |  |  |  |
| **9. Identity (Solidarity)** | -.03 | .01 | .06 | -.05 | .19 | .17 | .06 | .06 | — |  |  |  |  |  |  |  |  |  |  |
| **10. Identity (Satisfaction)** | .06 | .08 | -.22 | .08 | .20 | .59 | .36 | -.11 | .42 | — |  |  |  |  |  |  |  |  |  |
| **11. Identity (Centrality)** | .02 | -.08 | .22 | .01 | .07 | -.03 | .02 | .20 | .41 | .16 | — |  |  |  |  |  |  |  |  |
| **12. Identity (Self-stereotype)** | -.05 | -.02 | .08 | -.06 | .17 | .07 | .01 | .01 | .43 | .17 | .30 | — |  |  |  |  |  |  |  |
| **13. Identity (Homogeneity)** | -.10 | -.01 | .14 | -.04 | .09 | .01 | .05 | .09 | .33 | .13 | .32 | .65 | — |  |  |  |  |  |  |
| **14. Stigma** | .15 | -.13 | .14 | .11 | -.05 | -.08 | -.04 | .16 | .27 | .07 | .32 | .16 | .17 | — |  |  |  |  |  |
| **15. Loneliness** | .03 | -.48 | .43 | .37 | -.53 | -.30 | -.26 | .55 | -.02 | -.23 | .13 | -.01 | .07 | .18 | — |  |  |  |  |
| **16. Desire for a Partner** | -.13 | .05 | .32 | -.21 | -.06 | -.55 | -.32 | .06 | -.10 | -.61 | .06 | .10 | .08 | .02 | .15 | — |  |  |  |
| **17. Autonomy** | .07 | .30 | -.11 | -.23 | .34 | .19 | .15 | -.32 | .22 | .21 | .12 | .20 | .12 | -.01 | -.30 | .00 | — |  |  |
| **18. Commitment to Singlehood** | .17 | -.04 | -.26 | .19 | .15 | .54 | .39 | -.08 | .22 | .66 | .15 | .00 | .04 | .12 | -.14 | -.67 | .11 | — |  |
| **19. Positive Solitude** | .05 | .10 | -.10 | .02 | .15 | .26 | .18 | -.10 | .23 | .40 | .10 | .09 | .07 | .10 | -.15 | -.20 | .41 | .35 | — |

*Note.* All correlations above *r* = +/- 0.11 are significant at *p* < .05. Status Satisfaction = Relationship Status Satisfaction.

**Table 2b. Correlation Tables of Primary Variables for Partnered Individuals Only**

| **Variables** | **1** | **2** | **3** | **4** | **5** | **6** | **7** | **8** | **9** | **10** | **11** | **12** | **13** | **14** | **15** | **16** | **17** | **18** | **19** | **20** | **21** |
| --- | --- | --- | --- | --- | --- | --- | --- | --- | --- | --- | --- | --- | --- | --- | --- | --- | --- | --- | --- | --- | --- |
| **1. Age** | — |  |  |  |  |  |  |  |  |  |  |  |  |  |  |  |  |  |  |  |  |
| **2. Extraversion** | .03 | — |  |  |  |  |  |  |  |  |  |  |  |  |  |  |  |  |  |  |  |
| **3. Anxious Attachment** | -.33 | -.20 | — |  |  |  |  |  |  |  |  |  |  |  |  |  |  |  |  |  |  |
| **4. Avoidant Attachment** | -.02 | -.40 | .13 | — |  |  |  |  |  |  |  |  |  |  |  |  |  |  |  |  |  |
| **5. Life Satisfaction** | .13 | .42 | -.25 | -.27 | — |  |  |  |  |  |  |  |  |  |  |  |  |  |  |  |  |
| **6. Status Satisfaction** | -.14 | .17 | -.14 | -.31 | .44 | — |  |  |  |  |  |  |  |  |  |  |  |  |  |  |  |
| **7. Sexual Satisfaction** | -.18 | .25 | -.10 | -.21 | .41 | .45 | — |  |  |  |  |  |  |  |  |  |  |  |  |  |  |
| **8. Stress** | -.21 | -.45 | .53 | .23 | -.56 | -.24 | -.29 | — |  |  |  |  |  |  |  |  |  |  |  |  |  |
| **9. Identity (Solidarity)** | -.07 | .16 | .10 | -.18 | .20 | .25 | .28 | -.06 | — |  |  |  |  |  |  |  |  |  |  |  |  |
| **10. Identity (Satisfaction)** | -.12 | .21 | .05 | -.27 | .30 | .49 | .44 | -.12 | .59 | — |  |  |  |  |  |  |  |  |  |  |  |
| **11. Identity (Centrality)** | .01 | .16 | .14 | -.12 | .22 | .19 | .26 | .00 | .41 | .50 | — |  |  |  |  |  |  |  |  |  |  |
| **12. Identity (Self-stereotype)** | -.02 | .25 | .04 | -.16 | .27 | .23 | .33 | -.13 | .52 | .47 | .49 | — |  |  |  |  |  |  |  |  |  |
| **13. Identity (Homogeneity)** | -.06 | .22 | .06 | -.01 | .21 | .10 | .23 | -.09 | .42 | .33 | .40 | .63 | — |  |  |  |  |  |  |  |  |
| **14. Investment (Satisfaction)** | -.12 | .15 | -.14 | -.23 | .40 | .75 | .59 | -.21 | .33 | .62 | .30 | .32 | .20 | — |  |  |  |  |  |  |  |
| **15. Investment (Alternative)** | -.02 | .14 | .05 | .05 | .05 | -.17 | .07 | -.04 | .06 | -.05 | .01 | .11 | .15 | -.07 | — |  |  |  |  |  |  |
| **16. Investment (Size)** | -.03 | .01 | .06 | -.10 | .15 | .37 | .26 | .03 | .28 | .47 | .41 | .25 | .19 | .51 | -.06 | — |  |  |  |  |  |
| **17. Investment (Commitment)** | -.04 | .10 | -.11 | -.20 | .32 | .63 | .45 | -.15 | .29 | .59 | .29 | .28 | .12 | .81 | -.15 | .59 | — |  |  |  |  |
| **18. Stigma** | .00 | -.03 | .10 | .04 | -.04 | -.14 | -.07 | .09 | -.03 | -.05 | .00 | .02 | .04 | -.12 | .08 | -.10 | -.11 | — |  |  |  |
| **19. Loneliness** | -.17 | -.43 | .55 | .42 | -.44 | -.31 | -.28 | .57 | -.10 | -.14 | -.03 | -.09 | -.05 | -.30 | .04 | .01 | -.21 | .16 | — |  |  |
| **20. Autonomy** | -.14 | .35 | -.09 | -.22 | .30 | .17 | .30 | -.32 | .11 | .19 | .05 | .07 | .09 | .23 | .11 | .02 | .11 | .03 | -.26 | — |  |
| **21. Positive Solitude** | .02 | .20 | -.12 | .07 | .10 | -.02 | .13 | -.19 | .05 | .05 | .03 | .08 | .10 | -.01 | .14 | .01 | .03 | .00 | -.11 | .21 | — |

*Note*. All correlations equal to or higher than *r* = .10 is significant at *p* < .05

# **Appendix D: Supplemental Analyses: Confirmatory Factor Analysis with Avoidant Attachment**

Due to poor reliability of the six-item attachment avoidance subscale from the Experiences in Close Relationships scale, we performed a Confirmatory Factor Analysis (CFA) to assess item loadings. We conducted these analyses separately for single and partnered participants. Our approach primarily involved two steps: first, testing the model fit of the CFA using all six items, and then performing a second CFA after removing items with poor loadings. Finally, we re-ran the t-test analysis comparing conditions on avoidant attachment to determine if our initial null findings would change.

**Confirmatory Factor Analysis with Full and Reduced Attachment Avoidance Subscale**

**Single Participants.** For single participants, our initial CFA using all six attachment avoidance items, which revealed poor overall model fit (CFI = 0.59, TLI = 0.31, RMSEA = 0.24). Items 3 (loading = 0.189) and 11 (loading = 0.265; see Table S3) demonstrated the weakest loadings, so we removed these items and re-ran the CFA. The revised model showed improved fit indices (CFI = 0.95, TLI = 0.84, RMSEA = 0.14). Using a new composite comprised of items 1, 5, 7, and 9, we conducted a t-test comparing attachment avoidance between single participants in the singles-only and general conditions to examine whether results would change. Consistent with our original findings, we observed no significant difference (*t*(749) = −0.04, *p* = .97) in attachment avoidance across condition.

**Partnered Participants.** For partnered participants, the initial Confirmatory Factor Analysis (CFA) incorporating all six attachment avoidance items showed relatively poor model fit (CFI = 0.66, TLI = 0.43, RMSEA = 0.21). Items 1 (loading = 0.204) and 5 (loading = 0.144) exhibited the weakest loadings. After removing these items and rerunning the CFA with only items 3, 7, 9, and 11, we observed an improved model fit (CFI = 0.99, TLI = 0.98, RMSEA = 0.05). When we conducted the t-test using this new four-item composite, the results remained consistent, showing no significant difference in attachment avoidance between the partner-only and general partner conditions (*t*(812) = −1.27, *p* = .20).

Overall, while the reliability of six-item the attachment avoidance subscale is relatively low, adjusting the item composites for improved reliability does not change the final results such that for both single and partnered individuals, no differences were observed between the relationship-status specific and general conditions.

| **Table S3. Item Loadings from Confirmatory Factor Analysis of Avoidant Attachment Subscale** | | | | |
| --- | --- | --- | --- | --- |
|  | **Item Loadings (all items)** | | **Item Loadings (dropped)** | |
| **Items** | Single | Partnered | Single | Partnered |
| 1. It helps to turn to close others in times of need. (–) | 0.688 | 0.204 | 0.697 | – |
| 3. I want to get close to others, but I keep pulling back. | 0.189 | 0.595 | – | 0.616 |
| 5. I turn to close others for many things, including comfort and reassurance. (–) | 0.632 | 0.144 | 0.72 | – |
| 7. I try to avoid getting too close to others. | 0.372 | 0.733 | – | 0.72 |
| 9. I usually discuss my problems and concerns with close others. (–) | 0.715 | 0.378 | 0.674 | 0.328 |
| 11. I am nervous when others get too close to me. | 0.265 | 0.699 | – | 0.723 |
| *Note*. (–) indicates a reverse-scored item. Results show item loadings from the CFA including all items as well as the second CFA after dropping poorly loading items. | | | | |
